# Genome-Wide DNA Methylation Analysis of Soybean *Curled-Cotyledons* Mutant and Functional Evaluation of a Homeodomain-Leucine Zipper (HD-Zip) I Gene *GmHDZ20*

**DOI:** 10.3389/fpls.2020.593999

**Published:** 2021-01-11

**Authors:** Hui Yang, Zhongyi Yang, Zhuozhuo Mao, Yali Li, Dezhou Hu, Xiao Li, Guixia Shi, Fang Huang, Baohui Liu, Fanjiang Kong, Deyue Yu

**Affiliations:** ^1^Innovative Center of Molecular Genetics and Evolution, School of Life Sciences, Guangzhou University, Guangzhou, China; ^2^National Center for Soybean Improvement, National Key Laboratory of Crop Genetics and Germplasm Enhancement, Jiangsu Collaborative Innovation Center for Modern Crop Production, Nanjing Agricultural University, Nanjing, China; ^3^Institute of Industrial Crops, Henan Academy of Agricultural Sciences, Zhengzhou, China

**Keywords:** soybean, *curled-cotyledon*, mutant, DNA methylation, *GmHDZ20*

## Abstract

DNA methylation is a major, conserved epigenetic modification that influences many biological processes. Cotyledons are specialized tissues that provide nutrition for seedlings at the early developmental stage. To investigate the patterns of genomic DNA methylation of germinated cotyledons in soybean (*Glycine max*) and its effect on cotyledon development, we performed a genome-wide comparative analysis of DNA methylation between the soybean *curled-cotyledons* (*cco*) mutant, which has abnormal cotyledons, and its corresponding wild type (WT) by whole-genome bisulfite sequencing. The *cco* mutant was methylated at more sites but at a slightly lower level overall than the WT on the whole-genome level. A total of 46 CG-, 92 CHG-, and 9723 CHH- (H = A, C, or T) differentially methylated genes (DMGs) were identified in cotyledons. Notably, hypomethylated CHH-DMGs were enriched in the gene ontology term “sequence-specific DNA binding transcription factor activity.” We selected a DMG encoding a homeodomain-leucine zipper (HD-Zip) I subgroup transcription factor (GmHDZ20) for further functional characterization. GmHDZ20 localized to the nucleus and was highly expressed in leaf and cotyledon tissues. Constitutive expression of *GmHDZ20* in *Arabidopsis thaliana* led to serrated rosette leaves, shorter siliques, and reduced seed number per silique. A yeast two-hybrid assay revealed that GmHDZ20 physically interacted with three proteins associated with multiple aspects of plant growth. Collectively, our results provide a comprehensive study of soybean DNA methylation in normal and aberrant cotyledons, which will be useful for the identification of specific DMGs that participate in cotyledon development, and also provide a foundation for future in-depth functional study of *GmHDZ20* in soybean.

## Introduction

Cotyledons have critical functions during seed germination and seedling development as they serve as the main storage organ and also as the first photosynthetic organ to ensure seedling establishment, survival, and subsequent growth (Chandler, 2008). Studies in different plant species have indicated that removal of cotyledons at early developmental stages led to delayed seedling growth and flowering time and reduced biomass (Bisognin et al., 2005; Hanley and Fegan, 2007; Zhang et al., 2008; Wang et al., 2019). In *Arabidopsis thaliana*, loss-of-function mutations in *CUP-SHAPED COTYLEDON 1* (*CUC1*) and *CUC2* result in fused cotyledons and a reduced number of ovules (Aida et al., 1997; Cucinotta et al., 2018). Notably, hybrids with larger cotyledons are superior to their parents in terms of activating photosynthesis and auxin pathway genes in cotyledons (Fujimoto et al., 2012; Wang et al., 2019). These results suggest that the growth status of cotyledons is critical for seedling establishment and development, which might ultimately determine crop yield and quality. Soybean [*Glycine max* (L.) Merr.] is a major source of protein and oil for humans and animals worldwide (Graham and Vance, 2003). We reasoned that the study of cotyledon-deficient mutants could provide valuable information for crop breeding and improvement.

DNA cytosine methylation, which plays an essential role in plant growth and development, is a key aspect of epigenetic modification and a basic form of DNA covalent modification (Henderson and Jacobsen, 2007). Cytosine methylation affects plant morphology, stability, differentiation, and development through the regulation of gene expression, transposon silencing, and chromatin structure (Bucher et al., 2012; Gonzalez and Li, 2012). The dynamic processes of DNA methylation consist of *de novo* methylation, maintenance of methylation, and active demethylation, which are catalyzed by various enzymes of different regulatory pathways (Zhang et al., 2018). In plant DNA, cytosines are typically methylated in three sequence contexts, CG, CHG, and CHH (H represents A, T, or C) (Law and Jacobsen, 2010). CG methylation is predominantly maintained by DNA METHYLTRANSFERASE 1 (MET1) and CHG methylation is maintained by CHROMOMETHYLASE 2 and 3 (CMT2 and CMT3) (Zhang et al., 2010). The maintenance of CHH methylation is catalyzed by CMT2 or DOMAINS REARRANGED METHYLTRANSFERASE 2 (DRM2) depending on the genomic region (Zhang et al., 2010). DNA methylation especially occurs in intergenic regions where it limits transcription and proliferation of transposable elements (TEs) (Lippman et al., 2004). Gene-related DNA methylation in plants can occur in promoter regions or within the transcribed gene body (Zhang et al., 2018). DNA methylation in the promoter often affects gene transcription by silencing the nearby TE and other repeats, and gene body methylation may be associated with highly expressed genes (Zhang et al., 2018). Studies suggest that TE and gene body methylation are controlled by different mechanisms and regulations (Takuno and Gaut, 2013). An et al. (2017) revealed that DNA methylation undergoes dynamic changes during seed maturation, and CHH methylation levels in soybean cotyledons changed from 6% at the early stage S2 to 10 and 11% at the late stages S6 and S8, respectively. Song et al. (2013) analyzed the DNA methylation status in soybean roots, stems, leaves, and cotyledons of developing seedlings and demonstrated that the small RNA (smRNA) abundance was roughly positively correlated with hypermethylated regions but negatively related to hypomethylated regions in cotyledons. These studies suggest a relationship between DNA methylation and soybean cotyledon development.

Homeobox genes, which encode key transcription factors regulating biological growth and development, were first identified in *Drosophila* and further discovered in diverse organisms including plants (Schena and Davis, 1992; Mukherjee et al., 2009). Homeobox genes encode a conserved DNA-binding domain named homeodomain (HD) (Nam and Nei, 2005; Mukherjee et al., 2009). In plant genomes, homeobox genes constitute a large gene family (Chan et al., 1998; Bhattacharjee et al., 2015; Khan et al., 2018; Que et al., 2018; Li et al., 2020). *KNOTTED1* from maize (*Zea mays*) was the first homeobox gene cloned in plants (Vollbrecht et al., 1991). In plants, homeobox proteins can be divided into six subfamilies based on their HD domain and other characteristic motifs, including homeodomain-leucine-zipper (HD-Zip), KNOTTED1-like homeobox (KNOX), homeodomain-finger (PHD finger), Bell domain (Bell), Wuschel-related homeobox (WOX), and zinc finger-homeobox domain (ZF-HD) (Chan et al., 1998; Ariel et al., 2007). Among them, the HD-Zip gene family is specific to plants and is involved in many processes of growth and development (Ariel et al., 2007; Chen et al., 2019; Cai et al., 2020). HD-Zip proteins contain a N-terminal HD and a leucine motif (Ariel et al., 2007). In *Arabidopsis*, HD-Zip genes were classified into four subgroups according to their distribution and combination of additional domains (Ariel et al., 2007). HD-Zip I proteins in various species are involved in organ growth, abiotic stress, and auxin and light signaling pathways (Ariel et al., 2007). HD-Zip II genes are associated with light and hormone responses (Sawa et al., 2002; Sessa et al., 2005). HD-Zip III genes regulate the differentiation of apical meristems, embryogenesis, leaf polarity establishment, lateral organ development, and vascular bundle formation (Mattsson et al., 2003; Prigge et al., 2005). HD-Zip IV proteins play a key role in anthocyanin accumulation, epidermal cell differentiation, trichome formation, and root and cuticle development (Nakamura et al., 2006; Ariel et al., 2007). Chen et al. (2014) identified 88 HD-Zip genes in the soybean genome; however, the functions of most of these genes remain unclear.

In this research, we investigated the genome-wide methylation patterns and differences at CG, CHG, and CHH sites of germinated cotyledons from the soybean *curled-cotyledons* (*cco*) mutant, which has abnormal cotyledons, and the wild type (WT) via whole-genome bisulfite sequencing (WGBS). Based on this analysis, a differentially methylated gene (DMG) encoding an HD-Zip transcription factor, *GmHDZ20*, was cloned and functionally studied in soybean and *Arabidopsis*. Our results demonstrate the importance of DNA methylation in the development of soybean cotyledons and points to roles for *GmHDZ20* in leaf morphology, silique length, and seed number.

## Materials and Methods

### Plant Materials, Growth Conditions, and Phenotyping

Soybean (*Glycine max*) homozygous *curled-cotyledon* (*cco*) mutant seeds were derived from sodium azide (NaN_3_) and ^60^Co γ ray mutagenesis of a cultivar Nannong 94-16 (which served as the soybean WT) (Yu et al., 2012; Shi et al., 2014), which were all provided by the Soybean Research Institute, Nanjing Agricultural University, China. Soybean seeds were grown under natural field conditions at Jiangpu Experimental Station, Nanjing Agricultural University, Nanjing, China. Ten individual plants of the WT and the *cco* mutant were used for phenotype examination. The days to maturity of soybean plants was recorded at the R8 stage (days from emergence to when 95% of pods had attained the mature color; Fehr et al., 1971). Plant height and 100-grain weight per plant were recorded at the R8 stage. Seed protein and oil contents were measured with a near-infrared seed analyzer.

The WT *Arabidopsis thaliana* (Columbia-0; Col-0) plant was used as a non-transgenic control. Seeds were grown in a growth room under 16/8 h light/dark, 23/22°C, with 70% relative humidity. WT *Arabidopsis* and four T_2_ *GmHDZ20* transgenic lines were used for phenotype investigation. Ten-day-old and three-week-old seedlings were used to examine the cotyledon and leaf morphology, respectively, with ten seedlings (each line). The length and seed number of siliques were measured with ten seedlings (each line), and five siliques (each seedling). All data are shown as the mean values ± standard deviation (SD). Statistical analyses were performed using Microsoft Excel with two-tailed, two-sample Student’s *t*-tests.

### Library Construction and Sequencing

Seeds of soybean WT and the *cco* mutant were grown in a growth chamber at 25°C with 60% relative humidity under a 16/8 h light/dark cycle. At 6 days after germination, cotyledons (one sample for each genotype, each sample was pooled with three plants.) were respectively collected for Whole-Genome Bisulfite Sequencing (WGBS). Genomic DNA was extracted using a DNAsecure Plant Kit (TianGen, Beijing, China). The quality of DNA samples was evaluated with agarose gel electrophoresis and a Nanodrop ND1000 spectrophotometer (Nanodrop, Wilmington, DE). The concentration of DNA samples was measured with a Qubit 2.0 fluorometer (Life Technologies, CA, United States). Then, DNA samples were randomly fragmented to 100-300 bp with a sonicator (Covaris S220, Thermo Fisher Scientific, United States). After end repair and 3′-terminal-A extension, cytosine-methylated sequencing adaptors were ligated to DNA fragments according to the manufacturer’s instructions (Illumina). Further, bisulfite conversion was conducted using EZ DNA Methylation-Gold Kit (Zymo Research, CA, United States), and the DNA library was generated by PCR amplification. The concentration and size of the DNA library were confirmed by a Qubit2.0 fluorometer (Life Technologies, CA, United States) and an Agilent 2100 biological analyzer (Agilent, CA, United States), respectively. Finally, paired-end sequencing was performed using the Illumina Hiseq2500 platform by Shanghai Lingen Biotechnology Co., Ltd. (Shanghai, China). An un-methylated chloroplast genome of soybean was used to determine the bisulfite non-conversion rate (Li et al., 2018).

### WGBS Data Analysis

The original image data were converted to raw sequence data via base calling software. After removal of adapters, low quality reads, and reads containing ambiguous bases (Ns), the remaining clean reads were mapped to the soybean Williams82 reference genome^1^ with BSMAP aligner allowing up to 2 mismatches (Xi and Li, 2009). Finally, uniquely mapped reads were used to further analyze cytosine methylation sites and methylation levels as previously described (Lister et al., 2008). The annotations of soybean genomic genes and TEs were obtained from SoyBase database^2^.

Differentially methylated regions (DMRs) between the WT and *cco* genomes were identified according to the previously reported method (Zhang et al., 2016b; Zheng et al., 2017). Only cytosines with high-quality sequencing (coverage ≥ 4) in a library were considered. DMRs were searched with sliding-window size = 200 bp and step size = 50 bp. The average DNA methylation level of each region was defined as the proportion of methylated cytosines among total cytosines (mCs/total Cs). DNA methylation levels of the WT and the *cco* mutant were compared pairwise with Fisher’s exact test and the *p* values were adjusted via the Benjamini-Hochberg method for multiple comparisons. Windows with an adjusted *p-*value < 0.01 and an over 2.5-fold change of the methylation level were retained for further analysis. In addition, the *p-*value of each cytosine in the selected regions was evaluated by Fisher’s exact test, and cytosines with *p-*value < 0.01 were regarded as a differentially methylated cytosines (DMCs). Finally, the regions containing at least 7 DMCs were defined as DMRs, and adjacent DMRs were combined if the gap was less than or equal to 100 bp. Genes containing significant DMRs in their functional regions (promoter, untranslated region (UTR), exon, or intron) were defined as differentially methylated genes (DMGs).

The Soybean Expression Atlas online database^3^ was used to analyze the expression of all DMGs (material: Williams 82; cotyledons of seedlings 6 days after imbibition). Gene Ontology (GO) was analyzed through the GO term enrichment tool of SoyBase database^4^ and Kyoto Encyclopedia of Genes and Genomes (KEGG) pathways were predicted by KOBAS 3.0 program^5^. All significantly enriched GO terms and KEGG pathways were evaluated based on *p-*value after FDR correction < 0.01.

### RNA-Seq Data Analysis From a Public Database

Whole-genome gene expression values of the WT and the *cco* mutant were downloaded from our previous RNA-Seq dataset (Shi et al., 2014), which are deposited in the Gene Expression Omnibus (GEO) Database with accession number GSE58354. The soybean pods at 7 days after fertilization (DAF) from WT and *cco* plants were sampled for RNA-Seq using the Illumina HiSeq2000 system. Highly confident genes, including 33,214 in the WT and 33,264 in the *cco* mutant, were used for correlation analysis of DNA methylation and gene expression.

### Isolation and Sequence Analysis of *GmHDZ20*

The coding sequence (CDS) of *GmHDZ20* was downloaded from Phytozome^6^ and amplified from the cotyledon cDNA of the *cco* mutant with specific primers (Supplementary Table 1) using the following cycling profile: 98°C for 2 min followed by 35 cycles of 98°C for 10 s, 55°C for 30 s, and 68°C for 1 min. The amplified product was further gel purified, cloned into the pMD19-T vector, and sequenced (Invitrogen, Shanghai, China).

The isoelectric point and protein molecular weight were predicted with the ProtParam tool^7^. Protein sequence alignment of GmHDZ20 and eight other plant HD-Zip proteins was performed by ClustalW in MEGA version 6.0 with default parameters and viewed with GeneDOC. A Neighbor-joining (NJ) phylogenetic tree was built using MEGA 6.0 with 1000 bootstrap replications. Promoter *cis*-acting elements of *GmHDZ20* were evaluated with the Plant CARE database^8^.

### Expression Analysis of *GmHDZ20*

Total RNA was extracted using Plant RNA Extract Kit (TianGen, Beijing, China) and cDNA was synthetized with HiScript II Q RT Super Mix kit (Vazyme, Nanjing, China). Quantitative real-time polymerase chain reaction (qRT-PCR) was used to analyze the transcript levels of *GmHDZ20* in soybean on the Roche Light Cycler 480 system (Roche, Germany) with PCR kit (Roche, Germany) following the parameters: 95°C for 1 min and 40 cycles of 95°C for 5 s, 60°C for 30 s, and 72°C for 30 s, followed by a final extension at 72°C for 10 min. The soybean endogenous gene *TUBULIN* (GenBank accession no. AY907703) was used as an internal reference, and the relative expression levels were calculated based on the 2^−ΔΔCt^ method (Livak and Schmittgen, 2001). The qRT-PCRs were performed with three biological and technical replicates. Semi-quantitative RT-PCR (SqPCR) was conducted to examine the transcript levels of *GmHDZ20* in *Arabidopsis* by using 2 × UniqueTM Taq Super Master Mix (Novogene, Tianjin, China). The PCR protocol was 94°C for 5 min and 35 cycles of 94°C for 10 s, 56°C for 20 s, and 72°C for 1 min, followed by a final extension at 72°C for 5 min. The *Arabidopsis TUBULIN* gene (*AT5G62690*) was used as an internal reference. All primers are listed in Supplementary Table 1.

### Subcellular Localization of GmHDZ20

The *GmHDZ20* CDS was PCR amplified from the cotyledon cDNA of the *cco* mutant and cloned into the pAN580 vector to generate the recombinant construct *35S:GmHDZ20-GFP*, and then transformed into *Arabidopsis* (Col-0) protoplast cells according to a previously described method (Yoo et al., 2007). The empty vector *35S:GFP* was used as a positive control. The fluorescence images were obtained by confocal laser-scanning microscopy (Leica TCS SP2, Mannheim, Germany). This experiment was performed three times.

### *Arabidopsis* Transformation

The CDS of *GmHDZ20* was cloned into the pMDC83 vector using Gateway Technology (Invitrogen, Shanghai, China) and then transformed into *Arabidopsis* with the floral dip method (Mara et al., 2010). Transgenic *Arabidopsis* plants expressing *GmHDZ20* were first screened on Murashige and Skoog (MS) medium with 40 mg/ml Hygromycin B and further examined by PCR and SqPCR analyses.

### Yeast Two-Hybrid Assay

The full-length and different truncated CDS fragments of *GmHDZ20* were amplified with primers listed in Supplementary Table 1 and cloned into the bait vector pGBKT7 (BD). To examine their self-activation in yeast cells, each bait construct and empty prey vector pGADT7 (AD) were co-transformed into the yeast strain Y2H-Gold. SD/-Leu/-Trp/-His/-Ade medium was used to evaluate the protein-protein interactions through the activation of reporter genes, and SD/-Leu/-Trp medium was used for selection. Based on this, the bait plasmid BD-GmHDZ20 without self-activation was further used for library screening according to Matchmaker Gold Yeast Two-Hybrid System (Clontech, United States).

The soybean leaf cDNA library was constructed with RNAs of the mature upper-third leaves (cultivar Kefeng No.1 at R6 stage) using CloneMiner II cDNA Library Construction Kit (Invitrogen), and then cloned into pGADT7-Rec2-DEST (Zhang et al., 2016a). The soybean pod cDNA library was constructed with RNAs of the green pods (cultivar Kefeng No.1) and then cloned into pGADT7-Rec2-DEST. These two cDNA libraries were co-transformed with the bait construct into Y2H-Gold yeast competent cells. The prey plasmids were extracted from clones growing on SD/-Leu/-Trp/-His/-Ade medium and verified by retransformation with the bait GmHDZ20.

## Results

### Phenotypic Analysis of the Soybean *cco* Mutant

**FIGURE 1:**
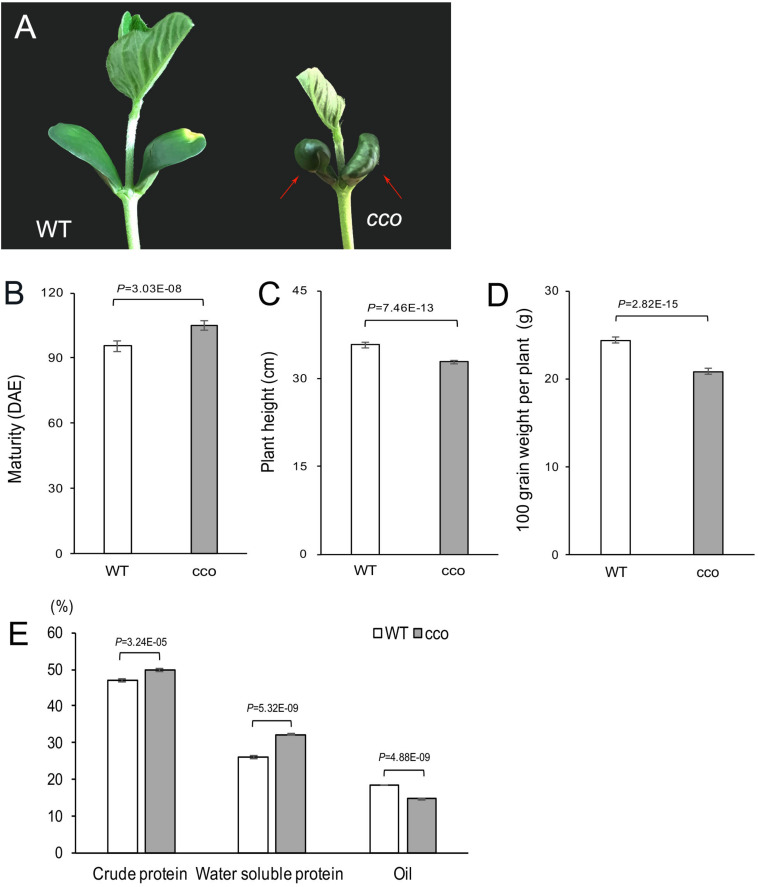
Phenotypic characterization of the soybean *cco* mutant and the WT. **(A)** Cotyledon morphology of 6-day-old seedlings; red arrows indicate curled cotyledons in the *cco* mutant. **(B)** Days to maturity of the WT and the *cco* mutant. Maturity was investigated at the R8 stage (days from emergence to 95% of pods attaining mature color); DAE, days after emergence. **(C)** Statistics of plant height at maturity stage. **(D)** Comparison of 100-grain weight per plant between the WT and the *cco* mutant. **(E)** Protein and oil contents in the WT and the *cco* mutant; the measurement was conducted with a near-infrared seed analyzer. Plants were grown in the field with natural long-day conditions. All data are shown as means ± SD (*n* = 10 plants). Two-tailed, paired-sample *t*-tests were used to generate the *p*-Values.

Besides the typical *curled-cotyledons* phenotype of the *cco* mutant (Figure 1A), the mutant also displayed significantly delayed maturity (Figure 1B), shorter plant height (Figure 1C), a smaller lateral root system (Yang et al., 2017), reduced 100-grain weight (Figure 1D), increased protein content (crude and water-soluble protein), and decreased oil content (Figure 1E) compared with the WT. Therefore, compound radiation-induced mutagenesis of the WT resulted in multiple defective phenotypes.

### Genome-Wide DNA Methylation Analysis of the WT and the *cco* Mutant

To uncover the extent of cytosine DNA methylation in the soybean *cco* genome, WGBS was applied on DNA isolated from cotyledons of the *cco* mutant and the WT. In total, 275,975,118 (WT) and 273,592,964 (*cco*) raw reads were obtained with higher than a 99% bisulfite conversion rate from two DNA library samples (Table 1). After trimming, 255,363,852 (WT) and 256,196,048 (*cco*) clean reads were generated (Table 1), of which 65.49% (WT) and 67.84% (*cco*) were uniquely mapped to the soybean Williams82 reference genome (Table 1). The average sequencing depth of the WT and *cco* genomes was 12.64× and 13.42×, respectively (Table 1).

**TABLE 1:** Summary of genome-wide methylation sequencing data.

Sample	Raw reads	Clean reads	Clean bases	Mapped reads	Mapped rate	Q30 (%)	Ave Depth	Conversion rate
WT	275,975,118	255,363,852	37,649,575,164	167,249,887	65.49%	96.21	12.64	99.22%
*cco*	273,592,964	256,196,048	37,817,221,803	173,792,521	67.84%	96.35	13.42	99.14%

**FIGURE 2:**
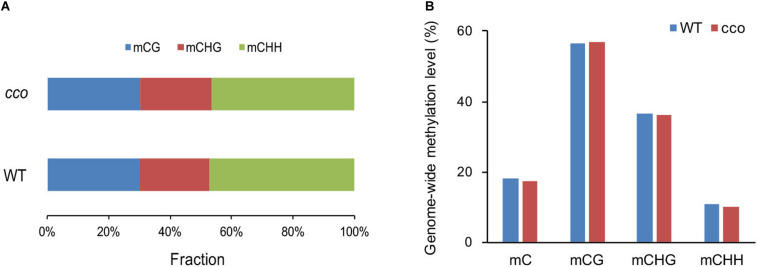
Genome-wide features of DNA methylation between the WT and the *cco* mutant. **(A)** Distribution of each context of methylated cytosines in the WT and *cco* genomes. **(B)** Genome-wide level of methylated cytosines (mC) and the mC level in three contexts (CG, CHG, and CHH) in the WT and *cco* genomes.

After removing sequencing depths < 4, a total of 357,036,725 and 364,453,643 methylated cytosine sites (mCs) were identified in the WT and *cco* genomes, respectively. Among them, the WT contains 107,348,557 methylated CGs (mCGs) (30.07% of all mCs), 81,313,556 mCHGs (22.77% of all mCs), and 168,374,612 mCHHs (47.16% of all mCs); the *cco* mutant has 110,112,002 mCGs (30.21% of all mCs), 85,000,478 mCHGs (23.32% of all mCs), and 169,341,163 mCHHs (46.46% of all mCs) (Figure 2A). Though the *cco* mutant has more methylated sites throughout its whole genome, its overall methylation level is slightly lower than that of the WT (Figure 2B). We detected 18.05% and 17.31% of cytosines in the WT and the *cco* mutant, respectively. However, the average level of methylated cytosines in the CG context (mCGs/CGs) in the *cco* mutant was higher than that in the WT, and the average levels of methylated cytosines in CHG (mCHGs/CHGs) and CHH (mCHHs/CHHs) contexts in the *cco* mutant were lower than those in the WT (Figure 2B). Clearly, the methylation level of CG and CHG sites is higher than that of CHH sites in both genotypes (Figure 2B).

**FIGURE 3:**
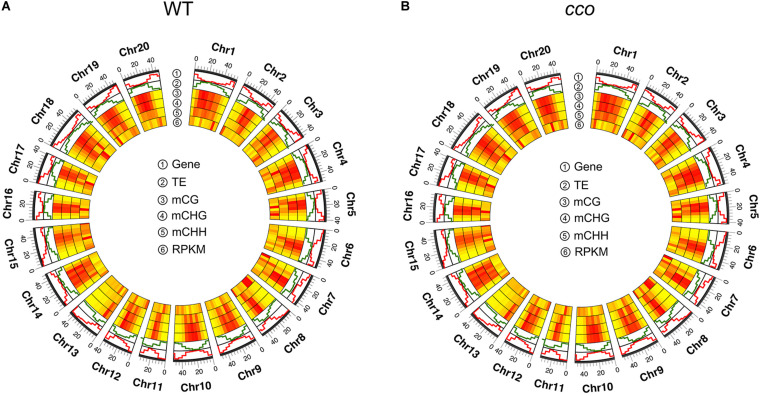
A circle plot of gene density, transposon density, DNA methylation level, and the transcript level for the WT **(A)** and the *cco* mutant **(B)**. Gene indicates “gene density;” TE indicates “transposon density;” mCG, mCHG, and mCHH indicate the methylated cytosine level in each context, respectively; RPKM (reads per kilobase million) indicates “gene expression level.” Data was plotted in 5-Mb windows on all soybean chromosomes. Bars with color from yellow to red indicate the methylation level or gene expression level from low to high, respectively. Pictures were drawn by TBtools.

Further, the correlations among DNA methylation, gene density, gene expression, and TE density on each chromosome were assessed. As shown in Figure 3, the DNA methylation level was unevenly distributed on the chromosomes in cotyledons of the WT and the *cco* mutant. On the whole, the WT and *cco* genomes showed a similar chromosomal DNA methylation pattern; the centromeric and pericentromeric regions of all 20 chromosomes were methylated at higher levels than non-pericentromeric regions in CG, CHG, and CHH contexts (Figures 3A,B). Also, the distributions indicated that gene-rich and TE-poor non-pericentromeric regions have lower methylation levels than the gene-poor and TE-rich centromeric and pericentromeric regions (Figures 3A,B), suggesting that DNA methylation might be positively associated with TE density and negatively associated with gene density. In addition, DNA methylation levels showed an opposite trend with gene expression levels on the majority of chromosomes (Figures 3A,B).

### Patterns of DNA Methylation in Gene Body and TE Regions

**FIGURE 4:**
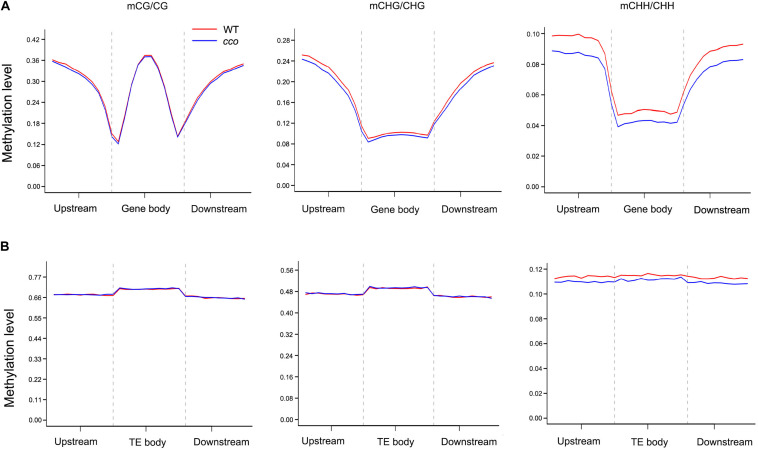
DNA methylation patterns of gene body and transposon regions in soybean cotyledons of the WT and the *cco* mutant. **(A)** Methylation levels of CG, CHG, and CHH contexts are displayed for each bin within gene body regions and their 2-kb up- and downstream regions. **(B)** Methylation levels of CG, CHG, and CHH contexts are displayed for each bin within transposons and their 2-kb up- and downstream regions. Gene body and transposon regions (with their flanking regions) were all equally divided into 20 bins. The methylation level of each bin is shown on the *Y*-axis.

We analyzed DNA methylation patterns in the 2-kb region upstream of the transcription start site (TSS), the gene body, and the 2-kb region downstream of the transcription termination site (TTS) of genes. As shown in Figure 4A, CG sites were methylated at relatively high levels, followed by CHG and CHH sites. For the 5′ and 3′ flanking regions, methylation levels of CG, CHG, and CHH sites increased rapidly as the distance from the TSS and TTS increased (Figure 4A). CHG and CHH sites had similar patterns with high methylation levels in 5′ and 3′ flanking regions, while low levels were observed in gene bodies (Figure 4A). By contrast, CG sites had high methylation levels in both gene body and flanking regions, while the levels dramatically dropped near TSSs and TTSs (Figure 4A). In general, CG, CHG, and CHH methylation patterns were consistent in the cotyledons of the WT and the *cco* mutant. However, in the *cco* mutant, CHG and CHH methylation levels were obviously lower than in the WT at gene bodies and their flanking regions (Figure 4A).

Analysis of TEs and the surrounding regions (2-kb upstream and downstream regions) showed that the methylation levels of CG and CHG sites in TEs were much higher than those in gene bodies, whereas CHH sites in TEs only had a slight increase in methylation level (Figure 4B). Different from the methylation patterns observed in gene bodies, methylation patterns of CG and CHG sites in TEs were similar with high methylation levels and relatively lower levels in their flanking regions (Figure 4B). Methylation levels of CHH sites were nearly equal in TEs and surrounding regions (Figure 4B). Overall, no obvious changes were observed in methylation patterns and methylation levels of CG or CHG sites between the WT and *cco* genomes (Figure 4B). CHH methylation levels of the *cco* mutant were apparently reduced compared with the WT (Figure 4B).

### Identification of Differentially Methylated Regions (DMRs) Between the WT and *cco* Genomes

**FIGURE 5:**
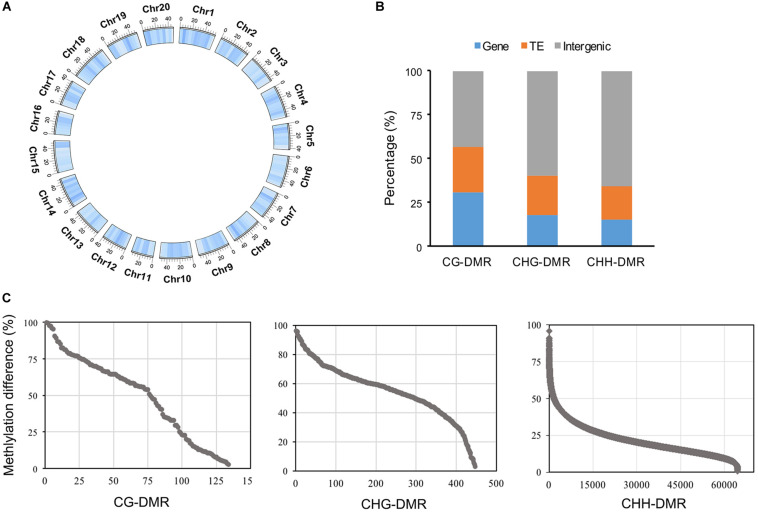
Characteristic features of DMRs in three cytosine contexts. **(A)** Genome-wide distribution of total DMRs with 5-Mb windows (darker blue indicates high density). **(B)** Genomic compositions of three different DMR contexts; gene region includes exons, introns, and UTRs. **(C)** Differences in the methylation level of three different DMR contexts.

We identified many more CHH-DMRs (64,470) between the WT and the *cco* mutant, and only a few CG-DMRs (134) and CHG-DMRs (447) were detected (Table 2 and Supplementary Table 2). Therefore, the length of CHH-DMRs was significantly longer than that of CG-DMRs and CHG-DMRs (Table 2). These CG-, CHG-, and CHH-DMRs were further classified into hypermethylated-DMRs (hyper-DMRs) and hypomethylated-DMRs (hypo-DMRs). CHG-DMRs and CHH-DMRs contained more hypo-DMRs than hyper-DMRs, while CG-DMRs had more hyper-DMRs than hypo-DMRs (Table 2). We then analyzed the distribution of total DMRs on the 20 chromosomes. As shown in Figure 5A, DMRs were distributed irregularly on chromosomes, but almost evenly throughout the genome. Subsequently, we compared the genomic compositions of DMRs in the three contexts and observed that CHG-DMRs and CHH-DMRs were similar with a higher proportion in intergenic regions, whereas more CG-DMRs existed in genic regions (Figure 5B and Supplementary Tables 3–5). There were 56.72 and 66.22% of CG-DMRs and CHG-DMRs with substantial (> 50%) differences in the methylation level within the DMRs (Figure 5C). As the average genome-wide methylation level of CHH sites was much lower than that of CG and CHG contexts, only a small proportion (2.61%) of CHH-DMRs had a differential methylation level greater than 50% (Figure 5C). These results indicate that the DMRs between WT and *cco* genomes in three sequence contexts have different characteristics.

**TABLE 2:** Statistics of the number and length of DMRs.

Type	Total	Hyper	Hypo	Length (bp)
CG-DMR	134	74	60	35,116
CHG-DMR	447	208	239	113,203
CHH-DMR	64,470	29,959	34,511	17,579,130

*Hyper, The DNA methylation level in the cco mutant was higher than that in the WT; Hypo, The DNA methylation level in the cco mutant was lower than that in the WT.*

### Analysis of Differentially Methylated Genes (DMGs) Between the WT and *cco* Genomes

**FIGURE 6:**
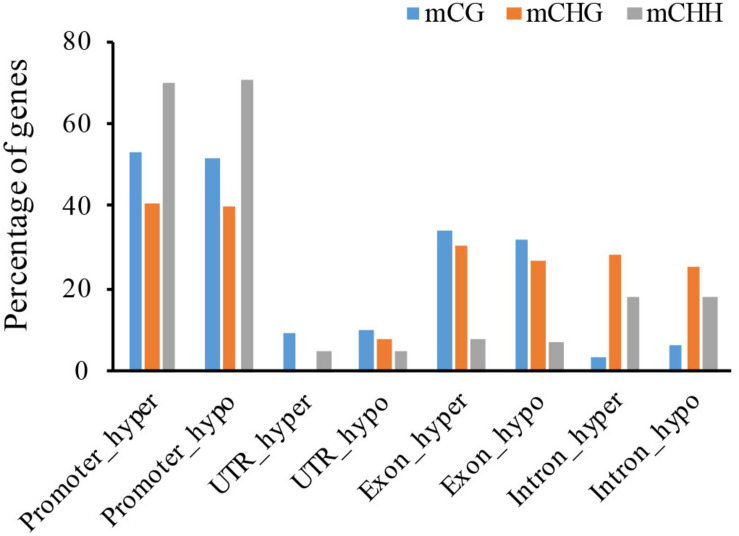
Distribution of hyper- and hypo-DMGs of three cytosine contexts in gene functional regions (promoter, UTR, exon, and intron).

To investigate the functional effects of DNA methylation variation, we analyzed genes located near DMRs. In total, we identified 46 CG-DMR, 92 CHG-DMR, and 9723 CHH-DMR non-repeat genes between the WT and the *cco* mutant in cotyledons (Table 3 and Supplementary Table 3), which revealed that CHH-DMGs might be mainly responsible for the morphological development of *cco* cotyledons. Similar with DMRs, there are more hypo-DMGs than hyper-DMGs in CHG and CHH contexts (Table 3). Further, Figure 6 and Supplementary Figure 1 show that about 70% of CHH-DMGs were hyper- and/or hypomethylated in the promoter region; there were more promoter- and exon-related DMGs in the CG context; while mCHG occupied a high percentage in promoter, exon, and intron regions.

**TABLE 3:** Number of hyper- and hypo-DMGs of three contexts in gene functional regions.

Type	Total (non-repeat)	Hyper					Hypo				
		Total	Promoter	UTR	Exon	Intron	Total	Promoter	UTR	Exon	Intron
mCG	46	32	17	3	11	1	31	16	3	10	2
mCHG	92	39	16	0	12	11	63	25	5	17	16
mCHH	9723	4666	3272	215	356	823	6751	4762	303	488	1198

**FIGURE 7:**
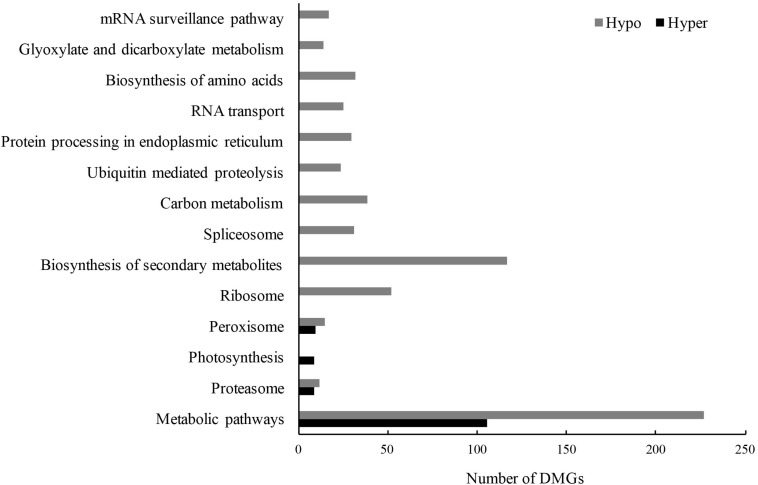
Significant KEGG enrichment pathways of hyper- and hypo-DMGs between the WT and the *cco* mutant (*p*-value after FDR correction < 0.01).

Subsequently, gene ontology (GO) and Kyoto Encyclopedia of Genes and Genomes (KEGG) analyses were used to explore the potential functions of these DMGs. Due to a smaller number of DMGs detected in CG and CHG contexts (Table 3), all DMGs from the three contexts were analyzed together. There were 3546 non-repeat hyper-DMGs and 6047 hypo-DMGs in total. Based on the online database Soybean Expression Atlas^9^, we obtained the expression data of all DMGs in cotyledons (6 days after imbibition) (Supplementary Table 6). After removal of the DMGs with lower expression level (TPM normalized less than 10), 926 hyper-DMGs and 1683 hypo-DMGs were retained and significantly enriched in 11 GO terms (corrected *p*-value < 0.01; FDR) (Table 4 and Supplementary Table 7). Of the 11 GO terms, 20 hyper-DMGs were associated with “iron-sulfur cluster binding” and “defense response” (Table 4 and Supplementary Table 7), and hypo-DMGs were involved in the following GO terms: “regulation of transcription,” “ubiquitin-dependent protein catabolic process,” “protein phosphorylation,” and “photorespiration” (Table 4 and Supplementary Table 7). More hypo-DMGs’ encoded products were related to “regulation of transcription” and “sequence-specific DNA binding transcription factor activity” (Table 4 and Supplementary Table 7), including WRKY, homeobox, MYB, NAC, GATA, bHLH, and bZIP transcription factors, indicating that transcription regulation was likely affected by DNA methylation in the early development of cotyledons. For example, the DMG *Glyma.04G223300* (*GmWRKY58*) plays a critical role in plant growth and flowering (Yang et al., 2016). *AtNAC2*, the homolog of the DMG *Glyma.13G280000*, is involved in embryogenesis (Kunieda et al., 2008), lateral root development, and salt stress response in *Arabidopsis* (He et al., 2005). Also, 14 significant KEGG pathways (corrected *p*-value < 0.01; FDR) were predicted, and plenty of hyper- and hypo-DMGs were enriched in “metabolic pathways” (Figure 7 and Supplementary Table 8). Additionally, the hyper- and hypo-DMGs participated in “proteasome,” “photosynthesis,” and “peroxisome” pathways. Compared with hyper-DMGs, hypo-DMGs were involved in more pathways like “ubiquitin-mediated proteolysis,” “RNA transport,” “biosynthesis of secondary metabolites,” and “biosynthesis of amino acids” (Figure 7 and Supplementary Table 8). In conclude, these DMGs might be involved in diverse biological processes to affect the phenotype of *cco* mutant.

**TABLE 4:** Significant GO terms of hyper- and hypo-DMGs between the WT and the *cco* mutant.

Type	ID	BG_item	DMG_item	FDR	Term
Hyper	GO:0051536	105	13	0.000415650	Iron-sulfur cluster binding
	GO:0006952	1116	7	0.007318558	Defense response
Hypo	GO:0010027	469	54	2.27E-07	Thylakoid membrane organization
	GO:0006355	4060	104	7.67E-07	Regulation of transcription, DNA-dependent
	GO:0003700	3846	81	9.86E-07	Sequence-specific DNA binding transcription factor activity
	GO:0006511	619	62	3.41E-06	Ubiquitin-dependent protein catabolic process
	GO:0006952	1116	15	1.41E-05	Defense response
	GO:0006468	2386	56	0.000135491	Protein phosphorylation
	GO:0019288	581	54	0.000470217	Isopentenyl diphosphate biosynthetic process, mevalonate-independent pathway
	GO:0006098	421	43	0.000581183	Pentose-phosphate shunt
	GO:0009853	337	35	0.003777406	Photorespiration

*DMG, differentially methylated gene; BG, background.*

### Identification and Characterization of Soybean *GmHDZ20*

**FIGURE 8:**
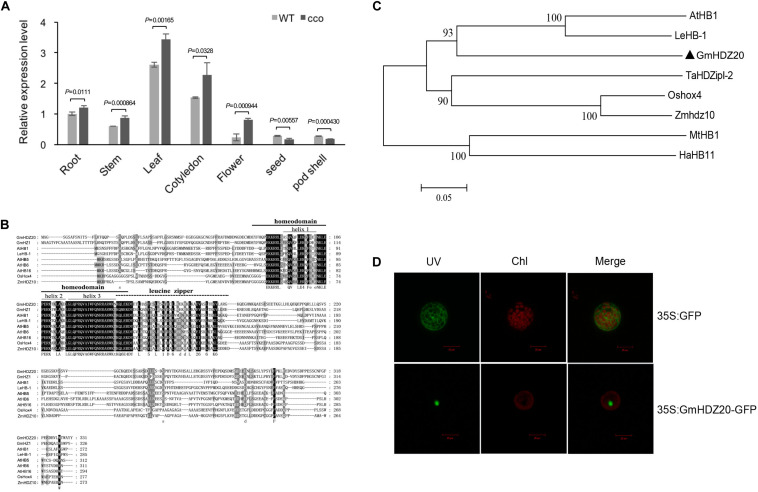
Sequence analysis, tissue-specific expression, and subcellular localization of *GmHDZ20*. **(A)** Tissue expression patterns of *GmHDZ20* in various organs in the WT and the *cco* mutant. *GmHDZ20* expression in root tissue was used as a control (expression value = 1). Means and SDs of three biological replications are shown. Statistical analysis was performed using paired-samples *t*-test (two-tail). **(B)** Sequence alignment of GmHDZ20 with other HD-Zip I homologs from different plant species. Black and gray indicate amino acid residues with different degrees of conservation. **(C)** Phylogenetic tree of *GmHDZ20* and other plant HD-Zip I genes. *GmHDZ20* homologous genes used are listed as follows: *GmHZ1* (AY823671) from soybean (*Glycine max*); *AtHB1* (AT3G01470), *AtHB5* (AT5G65310), *AtHB6* (AT2G22430), and *AtHB16* (AT4G40060) from *Arabidopsis thaliana*; *LeHB-1* (TC183162/Solyc02g086930) from tomato (*Lycopersicon esculentum*); *Oshox4* (AF145728) from rice (*Oryza sativa*); *Zmhdz10* (GRMZM2G041127) from maize (*Zea mays L*.); *TaHDZipI-2* (DQ353856) from wheat (*Triticum aestivum L*.); *MtHB1* (XM_003627463/Medtr8g026960) from *Medicago truncatula*; and *HaHB11* (DY923855) from sunflower (*Helianthus annuus*). **(D)** Subcellular localization of the GmHDZ20-GFP fusion protein. UV, Chl, and Merge represent GFP fluorescence, chlorophyll fluorescence, and a combination of the green and red images, respectively. A non-fused 35S:GFP construct served as a control. Scale bars: 20 μm. This experiment was performed three times with similar results.

Gene Ontology analysis indicated that many hypo-DMGs of the CHH context encode transcription factors (Table 4 and Supplementary Table 7), demonstrating that transcription regulation affected by methylation might be important for the formation of the curled cotyledons of the *cco* mutant. The promoter of *Glyma.05G030000* was hypomethylated in the *cco* mutant (Supplementary Table 3). *Glyma.05G030000* was named as *GmHDZ20* (Chen et al., 2014) and encodes a HD-Zip transcription factor. qRT-PCR was further used to analyze the tissue-specific expression of *GmHDZ20* in various tissues/organs of WT and *cco* plants at different developmental stages. *GmHDZ20* was expressed in all tissues examined, but its expression was highest in leaves and cotyledons, followed by roots, stems, and flowers, and lowest in seeds and pod shells (Figure 8A). Compared with the WT, the expression of *GmHDZ20* was significantly up-regulated in the *cco* mutant in all tissues except for seeds and pod shells (Figure 8A). Based on these results, *GmHDZ20* was selected for further functional study.

*GmHDZ20* contains three exons and two introns. The CDS of *GmHDZ20* was cloned via reverse transcription PCR (RT-PCR) from the cotyledons of the *cco* mutant (Supplementary Figure 2), which is 997 bp in length and encodes 311 amino acids along with an estimated protein mass of 37.14 kDa and a theoretical isoelectric point (PI) of 4.77. GmHDZ20 protein has conserved homeodomain and leucine zipper domains, and it belongs to the HD-Zip I subfamily (Figure 8B). Phylogenetic analysis indicated that GmHDZ20 was grouped together with AtHB1 (similarity of 44.88%) from *Arabidopsis* and LeHB-1 (similarity of 47.43%) from tomato (*Solanum lycopersicum*) (Figure 8C). Further, confocal images showed that GmHDZ20 is a nucleus-localized protein (Figure 8D).

Though GmHDZ20 may regulate target genes at the transcriptional level, its own activity might also be regulated by other protein factors. Therefore, we investigated the *cis*-elements in the promoter region of *GmHDZ20*. Based on PlantCARE database^10^, a 1500-bp fragment upstream of the ATG start codon of *GmHDZ20* was predicted to contain 36 *cis*-acting elements (Supplementary Table 9). Among them, 13 elements are involved in light responsiveness; 6 elements are related to hormone responsiveness including auxin, abscisic acid, gibberellin, salicylic acid, and methyl jasmonate; 5 elements are involved in stress responsiveness such as heat and drought; and 2 elements are associated with growth, one is required for endosperm gene expression and another is involved in circadian control (Supplementary Table 9). This suggests that *GmHDZ20* might be induced by several different regulatory factors.

### Overexpression of *GmHDZ20* in *Arabidopsis* Alters Leaf Morphology, Silique Length, and Seed Number

**FIGURE 9:**
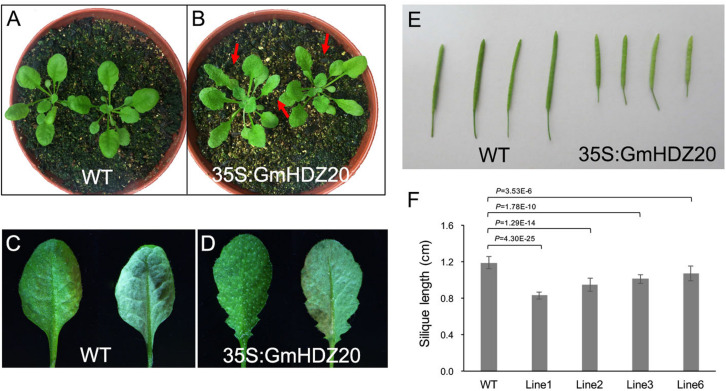
Phenotypic characterization of *35S:GmHDZ20 Arabidopsis* plants. **(A)** 3-week-old WT seedlings. **(B)** 3-week-old *GmHDZ20*-expressing transgenic seedlings. **(C)** WT adaxial and abaxial leaf. **(D)** Adaxial and abaxial leaf of the *GmHDZ20*-expressing transgenic plants. **(E)** Comparison of silique length between the WT and transgenic plants. **(F)** Statistics of silique length in WT and transgenic plants. Data are given as means ± SD (*n* = 10 seedlings). Two-tailed, paired-sample *t*-tests were used to generate the *p-*Values.

To further examine the role of *GmHDZ20*, we heterologously expressed the gene in *Arabidopsis* Col-0 plants driven by the Cauliflower mosaic virus 35S promoter and analyzed the morphology of the transgenic lines. Four *Arabidopsis* transgenic lines were used for phenotype investigation (Supplementary Figure 3). Compared with the *Arabidopsis* WT, all homozygous transgenic lines (3-week-old) from the T_2_ generation exhibited serrated rosette leaves (Figures 9A-D), but had no changes in cotyledon morphology at the early growth stage (10 days old) (Supplementary Figure 4A). At maturity, the silique length of *GmHDZ20*-expressing plants was significantly shorter than that of WT plants (Figures 9E,F). Further, the seed number per silique of the transgenic plants was significantly reduced compared to the WT (Table 5). However, the number of seeds per unit silique length was significantly lower in the WT than in the transgenic plants (Supplementary Figure 4B). These results suggested that *GmHDZ20* may regulate leaf morphology and affect silique development in *Arabidopsis*.

**TABLE 5:** Seed number per silique between WT and *GmHDZ20-*expressing *Arabidopsis* plants.

Genotype	35S:GmHDZ20				WT
	Line 1	Line 2	Line 3	Line 6	
Seed number per silique	43.1 ± 4.4	43.2 ± 5.6	42.7 ± 4.0	44.3 ± 5.0	48.5 ± 3.1
*p* values	1.46E-5	3.99E-4	5.76E-6	1.56E-3	

*Paired-samples t-test (two-tail) was used for statistical analysis.*

### Three Proteins Interacted With GmHDZ20 by Yeast Two-Hybrid Assay

To further study the molecular mechanism of GmHDZ20, a yeast two-hybrid (Y2H) assay was carried out to identify its interaction with proteins. First, to analyze the self-activation activity of GmHDZ20, its full-length and truncated CDS regions were tested by co-transforming each bait fusion pGBKT7 (BD) construct and empty prey vector pGADT7 (AD) into the yeast strain Y2H-Gold and then screened by selective medium.

**FIGURE 10:**
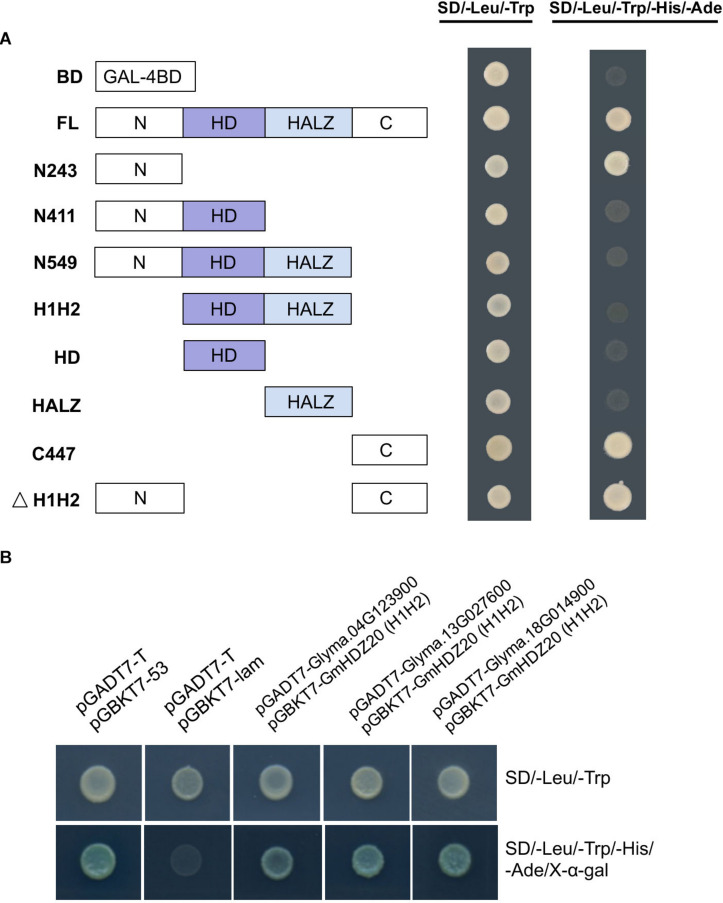
Identification of proteins that interacted with GmHDZ20 by the yeast two-hybrid assay. **(A)** Transactivation assay of different parts of *GmHDZ20* in yeast strain Y2H-Gold. BD, empty pGBKT7 vector; FL, full-length *GmHDZ20*; N243, GmHDZ20 N terminus with 243 nucleotides (1 to 81 residues); N411, N terminus with 411 nucleotides (1 to 137 residues); N549, N terminus with 549 nucleotides (1 to 183 residues); H1H2, homeodomain and leucine zipper domain with 306 nucleotides (82 to 183 residues); HD, homeodomain with 168 nucleotides (82 to 137 residues); HALZ, leucine zipper domain with 138 nucleotides (138 to 183 residues); C447, C terminus with 447 nucleotides (184 to 332 residues); ΔH1H2, N and C termini with 243 nucleotides (1 to 81 residues and 184 to 332 residues, respectively). **(B)** Three proteins interacted with GmHDZ20. Yeast two-hybrid assays were carried out between these three fusion AD proteins and the H1H2 fragment of GmHDZ20 (306 nucleotides; 82 to 183 residues) fused to BD bait. Positive: pGADT7-T and pGBKT7-53 plasmids; Negative: pGADT7-T and pGBKT7-lam plasmids.

The results indicated that the non-conservative N-terminal and C-terminal regions of GmHDZ20 have transcriptional activation function, while its homeodomain and leucine zipper domain could obviously suppress the transcriptional activation activity of GmHDZ20 (Figure 10A).

Therefore, the fusion plasmid BD-GmHDZ20 containing only the homeodomain and leucine zipper domain was used as bait to screen a soybean leaf and pod expression cDNA library generated in our lab. In this experiment, 25 colonies, including 15 different prey proteins, were identified by sequencing. After retesting, only three different prey proteins could be confirmed to interact with GmHDZ20 in yeast (Figure 10B). According to the functional annotation against the *Arabidopsis* genome, these proteins were identified as a ubiquitin-specific protease (*Glyma.13G027600*), a homeobox protein (*Glyma.18G014900*), and a heteroglycan glucosidase (*Glyma.04G123900*). In total, these results showed that GmHDZ20 may function together with other proteins and it requires support from future experimental evidence.

## Discussion

### The Soybean *cco* Mutant Has Multiple Defective Phenotypes

The cotyledons are the main nutrient storage tissue of soybean seed and supply nutrients to the seedling during vegetative growth (Chandler, 2008). The growth status of cotyledons in the early stage of seedling development is associated with plant resistance, yield, and quality in later stages of plant development (Chandler, 2008; Zhang et al., 2008). The *Arabidopsis* mutant *snowy cotyledon 1* (*sco1*), which has severely impaired chloroplast function, exhibits delayed seed germination and decreased plant growth and seed yield compared to the WT (Albrecht et al., 2006). In our study, in addition to curled cotyledons, the soybean *cco* mutant also showed several other defective phenotypes related to yield and quality (Figure 1). Shi et al. (2014) revealed that compared with the WT, the *cco* mutant showed abnormal embryogenesis beginning at the globular stage, resulting in malformed cotyledons and reduced germination rate of seeds. These phenotypic changes may be caused by gene mutations, or they might result from changes in cotyledon morphology that affects subsequent vegetative and reproductive growth of the plants.

### CHH Methylation May Play an Important Role in Soybean Cotyledon Growth

In recent years, significant progress has been made in the study of plant epigenetics (Song et al., 2013; Zhong et al., 2013; Shen et al., 2018; Zhang et al., 2018). DNA methylation is one of the most widely studied modifications in plant epigenetics (Niederhuth and Schmitz, 2014), which can affect transcriptional activity, morphological development, and agronomic traits (Manning et al., 2006; Miura et al., 2009; Quadrana et al., 2014). Moreover, DNA methylation is an important component of artificial selection in crop domestication in addition to genetic variation (Shen et al., 2018). Although the DNA methylome has been studied in different tissues in soybean (Song et al., 2013), genome-wide DNA methylation in germinated cotyledons is largely unexplored. In this research, we applied DNA methylation sequencing on cotyledons of the soybean *cco* mutant and its WT. The overall genome-wide DNA methylation level of the *cco* mutant is slightly lower than that of the WT (Figure 2A), but there are no significant changes in methylation at the CG, CHG, and CHH contexts. However, a large number of DMRs in CHH sites and a small number of DMRs in CG and CHG sites were present. A total of 134 CG-, 447 CHG-, and 64,470 CHH-DMRs were identified between the WT and *cco* genomes (Table 2), suggesting that these CHH-DMRs might be involved in regulating cotyledon development. This result is similar with a previous study on DNA methylation analysis of cotyledons during soybean seed maturation (An et al., 2017), which possibly indicates that DNA methylation, particularly CHH methylation, plays an important role in the development of both developing and germinated cotyledons. Shen et al. (2018) (Shen et al., 2018) identified 4248 DMRs in the process of soybean domestication (termed Dos-DMR) and 1164 DMRs in the improvement process (termed Imp-DMR) through analysis of WGBS data of 45 soybean accessions (including wild soybeans, landraces, and cultivars). Eight DMRs in our study overlapped with soybean Dos-DMRs or Imp-DMRs; among them, 3 CG-DMRs, 2 CHG-DMRs, and 2 CHH-DMRs were overlapped with Dos-DMRs, and 1 CG-DMR overlapped with an Imp-DMR, which may indicate that the genes in these DMRs might be important for soybean domestication or improvement.

### Hypomethylated CHH-DMGs Might Be Involved in Diverse Biological Processes

Within the DMRs, we respectively identified 46, 92, and 9723 non-repeat genes in CG, CHG, and CHH contexts between the WT and the *cco* mutant (Table 3 and Supplementary Table 3). Further, GO and KEGG analyses were used to detect some key processes potentially related to cotyledon development. Hypomethylated CHH-DMGs were enriched in diverse biological processes (Figure 7 and Table 4). For instance, the DMG *Glyma.09G095700*, encoding a *KNOTTED1*-like homeobox transcription factor, is homologous to *KNAT3* in *Arabidopsis* (Supplementary Table 10). *AtKNAT3* was previously reported to not only regulate embryo sac development, but also to modulate seed germination and early seedling development through the abscisic acid-mediated pathway (Kim et al., 2013). A *CONSTANS*-like (*COL*) DMG, *Glyma.06G059600*, hypomethylated in the promoter region, may have a similar function as its homolog *AtCOL4* (Supplementary Table 10). *Arabidopsis* COL4 is a flowering repressor in long days and short days and acts on the expression of *FLOWERING LOCUS T* (*FT*) and *FT*-like genes, as well as on *SUPPRESSOR OF OVEREXPRESSION OF CONSTANS 1* (*SOC1*) (Steinbach, 2019). The DMG *Glyma.10G165900* was predicted to encode a homolog of the MYB-related protein CELL DIVISION CYCLE 5 (CDC5) of *Arabidopsis* (Supplementary Table 10), which is essential for the G2/M phase transition of the cell cycle and may control the function of the shoot apical meristem by regulating the expression of *SHOOT MERISTEMLESS* (*STM*) and *WUSCHEL* (*WUS*) (Lin et al., 2007). *Glyma.06G164900* was annotated as an AUXIN RESPONSE FACTOR 2 (ARF2) (Supplementary Table 10); *arf2* mutants in *Arabidopsis* have a pleiotropic phenotype, including enlarged rosette leaves, reduced fertility, delayed senescence, hypocotyl elongation defects, enlarged seeds, and enlarged cotyledons (Ellis et al., 2005; Okushima et al., 2005; Schruff et al., 2006). CONSTITUTIVE PHOTOMORPHOGENIC9 (COP9) signalosome (Supplementary Table 10), a homolog of the DMG *Glyma.04G115900*, was recently reported to have a global genome-wide effect on methylation in *Arabidopsis*, and part of the pleiotropic phenotype of the *cop9* mutant might be due to global effects on DNA methylation (Tuller et al., 2019). S-adenosylmethionine is involved in ethylene, nicotianamine, and polyamine biosynthetic pathways and provides the methyl group for protein and DNA methylation reactions (Wang et al., 2002; Shyh-Chang et al., 2013). *Arabidopsis S-ADENOSYLMETHIONINE SYNTHETASE 2* (*AtSAM2*) (Supplementary Table 10), a homolog of the DMG *Glyma.10G144300*, interacts with the plasma membrane receptor-like kinase FERONIA (FER) (Supplementary Table 10) to suppress S-adenosylmethionine production and ethylene biosynthesis in *Arabidopsis* (Mao et al., 2015). Loss-of-function mutants of *fer* generate higher levels of S-adenosylmethionine and ethylene in plant tissues and display a dwarf phenotype (Mao et al., 2015). Taken together, we hypothesize that these DMGs mentioned here might directly or indirectly affect the phenotype of the *cco* mutant.

### *GmHDZ20* May Regulate Leaf Morphology, Silique Length, and Seed Number

Hypomethylated CHH-DMGs were enriched for those encoding transcription factors. Because transcription factors have a strong effect on transcriptional networks (Yamasaki et al., 2013), we hypothesized that some of these genes may play an important role in cotyledon or leaf development. Among these transcription factors, homeobox transcription factors regulate many aspects of biological growth and development (Mukherjee et al., 2009). The *Arabidopsis* homeobox gene *AtHB1*, a homolog of the CHH-DMG *Glyma.05G030000*, regulates cotyledon, leaf, and hypocotyl development in *Arabidopsis* (Aoyama et al., 1995; Capella et al., 2015). Thus, we focused on a HD-Zip protein-encoding gene, *GmHDZ20*, for further functional investigation. Several candidate *cis*-elements exist in the *GmHDZ20* promoter (Supplementary Table 9), in which methylation is decreased in the *cco* mutant (Supplementary Table 3). The hypomethylation in the promoter was associated with higher expression in cotyledons of the *cco* mutant than in the WT (Figure 8A); it appears that promoter DNA methylation inhibits *GmHDZ20* transcription.

HD-Zip transcription factors are plant-specific and have a wide range of regulatory roles in plant growth and development, such as organ development, meristem formation, and stress response (Ariel et al., 2007). For example, *Arabidopsis AtHB13* regulates the morphological development of cotyledons and leaves, and participates in sucrose signal transduction (Hanson et al., 2001). Rice (*Oryza sativa*) *OsSLI1*, induced by multiple abiotic stresses and exogenous abscisic acid, was demonstrated to be a transcriptional activator regulating stress-responsive gene expression and panicle development in rice (Huang et al., 2014). However, the function of most members of the soybean HD-Zip gene family is uncertain. GmHDZ20 showed close phylogenetic relationships with AtHB1 from *Arabidopsis* and LeHB-1 (Figure 8B), and thus may possess similar functions to its homologs in organ development (Aoyama et al., 1995; Lin et al., 2008; Capella et al., 2015). Tomato (*Solanum lycopersicum*) *LeHB-1* was reported to participate in floral organogenesis and fruit ripening (Lin et al., 2008).

The development of leaves and other lateral organs (such as flowers, pods, or seeds) is often controlled by the same key genes (Jeong et al., 2012; Zhao et al., 2017). Our current results show that heterologous expression of *GmHDZ20* not only affects the number of seeds per silique in *Arabidopsis*, but also changes the shape of rosette leaves (Figure 9 and Table 5). Therefore, *GmHDZ20* may have regulatory effects on leaf morphology and seed number per pod. However, no differences in cotyledon morphology were observed between *Arabidopsis* transgenic plants expressing *GmHDZ20* and WT plants. The T_0_ generation of a soybean *GmHDZ20* clustered regularly interspaced short palindromic repeats (CRISPR)/CRISPR-associated protein 9 (Cas9)-mediated knockout mutant has been obtained and will be used for further phenotype investigation.

In addition, GmHDZ20 physically interacted with three proteins in yeast cells (Figure 10B). *Glyma.13G027600* is homologous to *AT5G06600* (*UBP12*), which plays important roles in regulating root meristem development (An et al., 2018), plant innate immunity (Ewan et al., 2011), photoperiodic flowering (Cui et al., 2013), and jasmonic acid signaling (Jeong et al., 2017). The homologous gene *AT2G22430* (*HB6*) of *Glyma.18G014900* is a target of the protein phosphatase ABI1 and regulates hormone responses in *Arabidopsis* (Himmelbach et al., 2002). The heteroglycan glucosidase encoded by *Glyma.04G123900* is highly expressed in the embryo and endosperm of soybean seed based on Soybean Expression Atlas database, and GO enrichment indicated that *Glyma.04G123900* is involved in carbohydrate metabolic processes. Notably, *Glyma.13G027600* and *Glyma.04G123900* also showed significant differences in methylation levels in our WGBS data (Supplementary Table 3). Nevertheless, further experiments are needed to verify the physical interactions between these proteins and to investigate how they participate in the regulatory function of *GmHDZ20*.

## Data Availability Statement

All whole-genome bisulfite sequencing raw data were deposited into the Sequence Read Archive (SRA) database in NCBI under Accession Number PRJNA668884.

## Author Contributions

DY designed the research. HY and ZY conducted most of the experiments and analyzed the data. HY wrote the manuscript. DY, FH, BL, and FK revised the manuscript. ZM, YL, DH, XL, and GS assisted with the experiments. All authors contributed to the article and approved the submitted version.

## Conflict of Interest

The authors declare that the research was conducted in the absence of any commercial or financial relationships that could be construed as a potential conflict of interest.

## Footnotes

1 https://phytozome.jgi.doe.gov/pz/portal.html#!info?alias=Org_Gmax

2 https://www.soybase.org/

3 http://venanciogroup.uenf.br/resources/

4 https://soybase.org/goslimgraphic_v2/dashboard.php

5 http://kobas.cbi.pku.edu.cn/kobas3/?t=1

6 https://phytozome.jgi.doe.gov/pz/portal.html

7 http://web.expasy.org/protparam/

8 http://bioinformatics.psb.ugent.be/webtools/plantcare/html/

9 http://venanciogroup.uenf.br/resources/

10 http://bioinformatics.psb.ugent.be/webtools/plantcare/html/
