# Supplementary material for: Genome-Wide DNA Methylation Analysis of Soybean Curled-Cotyledons Mutant and Functional Evaluation of a Homeodomain-Leucine Zipper (HD-Zip) I Gene GmHDZ20
Source: Front Plant Sci. 2021 Jan 11;11:593999. doi: 10.3389/fpls.2020.593999 (PMC7830220; doi:10.3389/fpls.2020.593999)
Supplement: Supplementary file 2 [file Data_Sheet_2.pdf]

Genome-wide DNA methylation analysis of soybean curled-cotyledons mutant and functional evaluation of a homeodomain-leucine zipper (HD-Zip) I gene *GmHDZ20*

Hui Yang[†,*], Zhongyi Yang[†], Zhuozhuo Mao, Yali Li, Dezhou Hu, Xiao Li, Guixia Shi, Fang Huang, Baohui Liu, Fanjiang Kong and Deyue Yu[*]

[*] Correspondence:

Hui Yang: smashing_hui@163.com

Deyue Yu: dyyu@njau.edu.cn

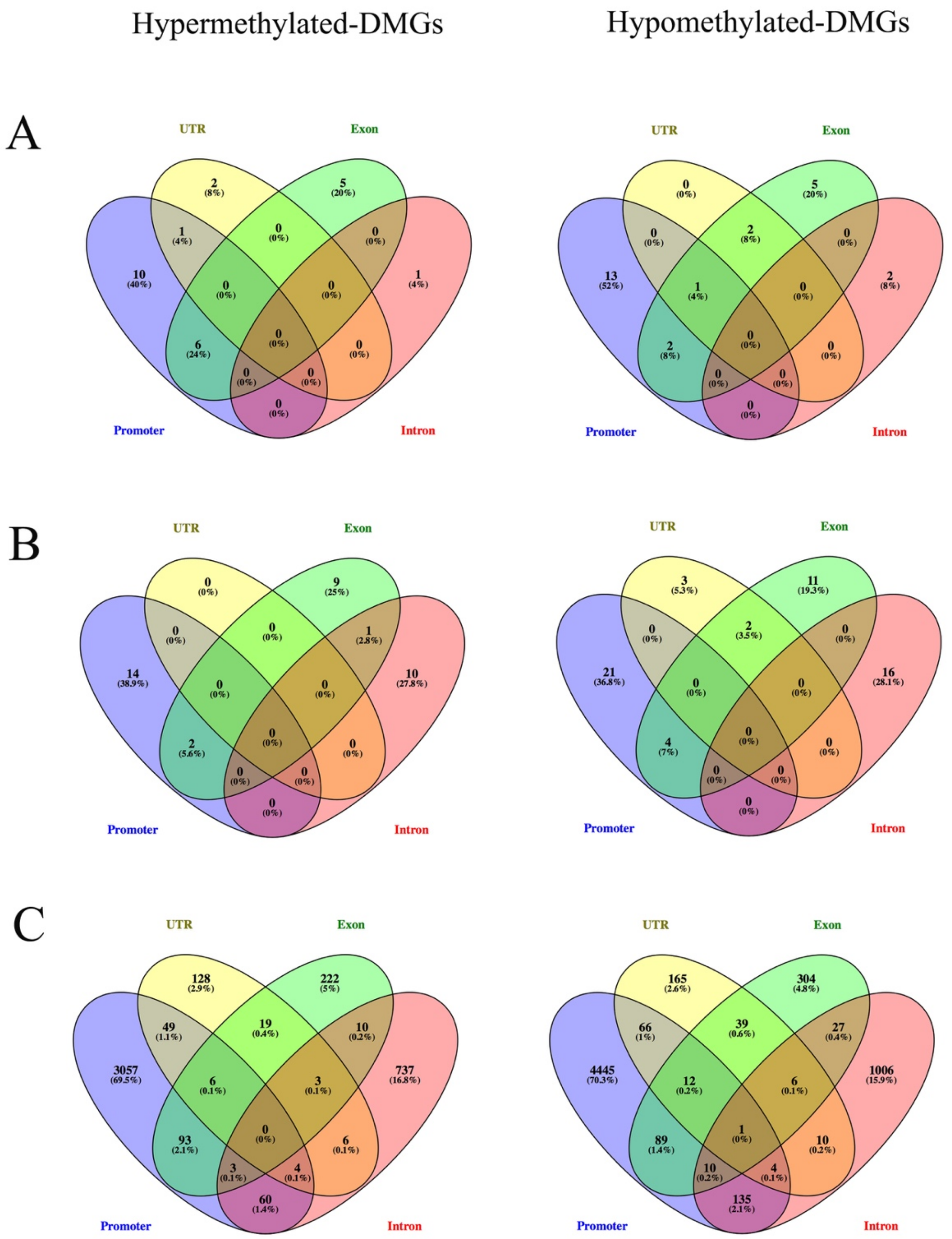

Supplementary Figure S1. Venn diagram of hyper- and hypo-DMGs in gene functional regions of the CG **(A)**, CHG **(B)**, and CHH **(C)** contexts.

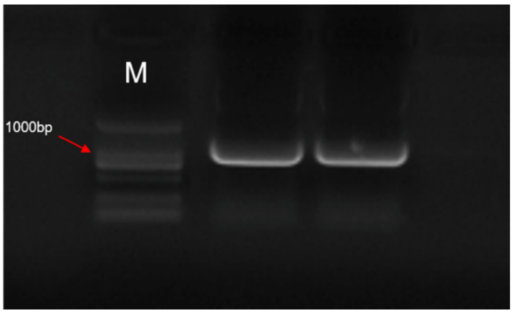

Supplementary Figure S2. Amplification of the *GmHDZ20* coding sequence. M: Trans2K DNA marker.

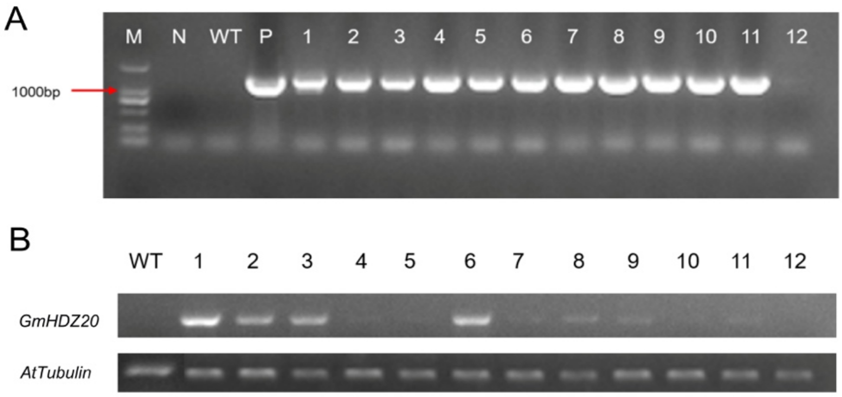

Supplementary Figure S3. Identification of T_2 transgenic *Arabidopsis* plants heterologously expressing *GmHDZ20*. **(A)** Transgenic plants were identified by amplifying *GmHDZ20* from the genomic DNA. **(B)** Semi-quantitative RT-PCR analysis of *GmHDZ20* expression in different transgenic lines. M: Trans2K DNA marker; P: positive control (recombinant plasmid); N: negative control (water); WT: Col-0 ecotype; Numerical code: different transgenic lines.

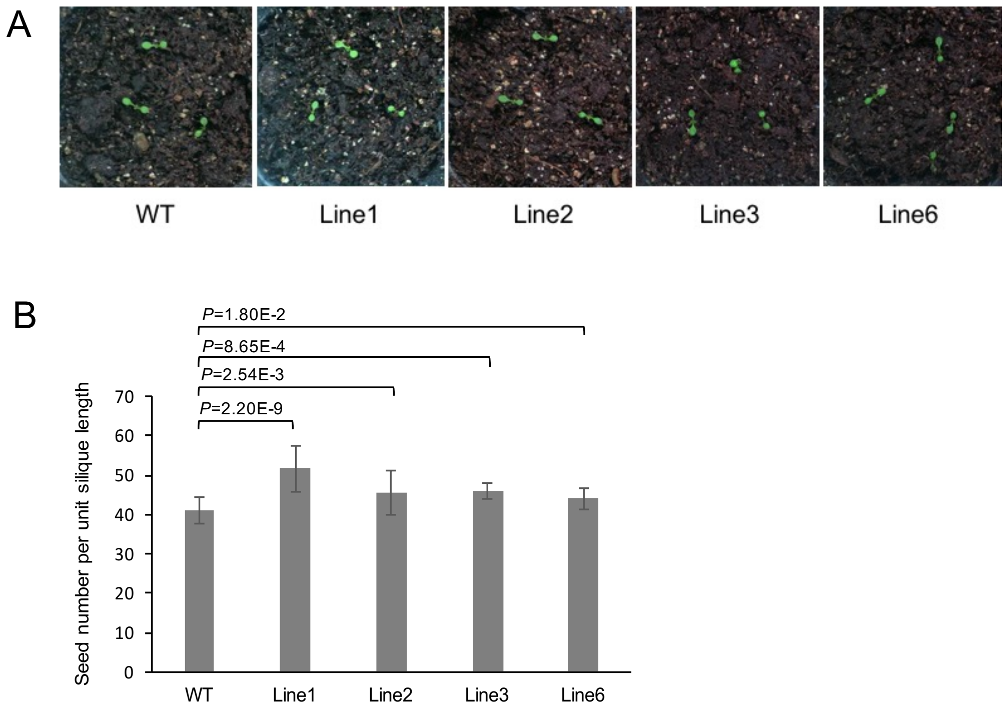

Supplementary Figure S4. Comparison of cotyledon morphology and seed number per unit silique length between *Arabidopsis* WT and *GmHDZ20* transgenic plants. **(A)** The cotyledons of 10-day-old seedlings. **(B)** Statistics of seed number per unit silique length. Data are given as means ± SD (n = 10 seedlings). Two-tailed, paired-sample *t*-test were used to generate the *p* values.

Supplementary Table S1 Primer pairs used for experiments

Gene Names	Sense primers (5'-3')	Antisense primers (5'-3')	Function
GmHDZ20	ACAACCCTCTTCTCAACCTCT	GGTCTTTGGCTAGTTTGGTCT	qRT-PCR
GmTubulin	GGAGTTCACAGAGGCAGA	CACTTACGCATCACATAGCA	qRT-PCR
AtTubulin	CTCAAGAGGTTCTCAGCAGTA	TCACCTTCTTCATCCGCAGTT	qRT-PCR
GmHDZ20	AAGTCCGGAGCTAGCTCTAGATGGCGG<br>GTAGTGGAAGTGC	GCCCTTGCTCACCATGGATCCGTAGTAA<br>GCCCAGGTCCAAAGG	Subcellular localization
GmHDZ20	GGGGACAAGTTTGTACAAAAAAGCAG<br>GCTATGGCGGGTAGTGGAAGTGC	GGGGACCACTTTGTACAAGAAAGCTGG<br>GTTTAGTAGTAAGCCCAGGTCCAAA	Transformation
GmHDZ20-FL	TCTCAGAGGAGGACCTGCATATGGCGG<br>GTAGTGGAAGTGC	CCGCTGCAGGTCGACGGATCTTAGTAG<br>TAAGCCCAGGTCCAAA	Transcriptional activation<br>activity analysis
N243	TCTCAGAGGAGGACCTGCATATGGCGG<br>GTAGTGGAAGTG	CCGCTGCAGGTCGACGGATCGGGTTGA<br>TGAAAGTACTCGTC	Transcriptional activation<br>activity analysis
N411	TCTCAGAGGAGGACCTGCATATGGCGG<br>GTAGTGGAAGTG	CCGCTGCAGGTCGACGGATCGTTCTTC<br>CACCGAGCTCTACG	Transcriptional activation<br>activity analysis
N549	TCTCAGAGGAGGACCTGCATATGGCGG<br>GTAGTGGAAGTGC	CCGCTGCAGGTCGACGGATCCCCTCTT<br>GCAAGCACCTTCTCAG	Transcriptional activation<br>activity analysis
H1H2	TCTCAGAGGAGGACCTGCATGAGAAG<br>AAGCGACGTCTCTCTGCG	CCGCTGCAGGTCGACGGATCCCCTCTT<br>GCAAGCACCTTCTCA	Transcriptional activation<br>activity analysis
HD	TCTCAGAGGAGGACCTGCATGAGAAG<br>AAGCGACGTCTCTCTG	CCGCTGCAGGTCGACGGATCGTTCTTC<br>CACCGAGCTCTACG	Transcriptional activation<br>activity analysis
HALZ	TCTCAGAGGAGGACCTGCATAAACAGC<br>TGGAGAAGGATTACGAGA	CCGCTGCAGGTCGACGGATCCCCTCTT<br>GCAAGCACCTTCTCA	Transcriptional activation<br>activity analysis
C447	TCTCAGAGGAGGACCTGCATAAACAA<br>GAGGGGCACATGAAGC	CCGCTGCAGGTCGACGGATCTTAGTAG<br>TAAGCCCAGGTCCAAAGG	Transcriptional activation<br>activity analysis
ΔH1H2-1	TCTCAGAGGAGGACCTGCATATGGCGG<br>GTAGTGGAAGTG	GCTTCATGTGCCCCTCTTGTTTGGGTTG<br>ATGAAAGTACTCGTC	Transcriptional activation<br>activity analysis
ΔH1H2-2	GACGAGTACTTTCATCAACCCAAACAA<br>GAGGGGCACATGAAGC	CCGCTGCAGGTCGACGGATCTTAGTAG<br>TAAGCCCAGGTCCAAAGG	Transcriptional activation<br>activity analysis
